# Distribution and Origin of Major, Trace and Rare Earth Elements in Wild Edible Mushrooms: Urban vs. Forest Areas

**DOI:** 10.3390/jof7121068

**Published:** 2021-12-12

**Authors:** Maja Ivanić, Martina Furdek Turk, Zdenko Tkalčec, Željka Fiket, Armin Mešić

**Affiliations:** Ruđer Bošković Institute, Division for Marine and Environmental Research, Bijenička 54, 10000 Zagreb, Croatia; mivanic@irb.hr (M.I.); mfurdek@irb.hr (M.F.T.); ztkalcec@irb.hr (Z.T.); armin.mesic@irb.hr (A.M.)

**Keywords:** mushrooms, trace elements, rare earth elements, urban soils, forest

## Abstract

This paper investigates the composition of major, trace, and rare earth elements in 15 different species of wild edible mushrooms and the possible effect of urban pollution on elemental uptake. The collected mushrooms include different species from the green areas of the city, exposed to urban pollution, and from the forests, with limited anthropogenic influence. Through a comprehensive approach that included the analysis of 46 elements, an attempt was made to expand knowledge about element uptake by mushroom fruiting bodies. The results showed a wide variability in the composition of mushrooms, suggesting a number of factors influencing their element uptake capacity. The data obtained do not indicate significant exposure to anthropogenic influences, regardless of sampling location. While major elements’ levels appear to be influenced more by species-specific affinities, this is not true for trace elements, whose levels presumably reflect the geochemical characteristics of the sampling site. However, the risk assessment showed that consumption of excessive amounts of the mushrooms studied, both from urban areas and from forests, may have adverse health effects.

## 1. Introduction

Mushrooms have been a part of the human diet for centuries. Nowadays, they are not only readily consumed, but they are also very popular in the category of healthy foods and delicacies. Beyond diets, mushrooms feature in some types of traditional medicine, but also as fractions in various medical supplements [1,2]. In Europe, they are also defined as “novel foods”, that is, foods that were not consumed to any significant extent before 1997.

Since food consumption is generally considered the most likely route of human exposure to heavy metals, the levels of individual metal species in mushrooms became of interest not only to the scientific community, but also to many regulatory agencies. Due to the increased intake of heavy metals and their persistence in the environment, these pollutants are among the most serious environmental problems. Their accumulation in soils as well as slow degradation processes enable their efficient transfer into living organisms, and consequently through the food chain to humans [3]. For this reason, the content of metals and metalloids in mushrooms, both wild and cultivated, should be continuously monitored.

Mushrooms are known to readily accumulate high concentrations of metals and metalloids, such as mercury, cadmium, lead, copper, arsenic, and radionuclides [4,5,6,7]. They take up elements from a substrate through an extensive mycelium, and their total content is influenced by both fungal and environmental factors [8,9,10]. In addition to species-specific affinity, the morphological part of the fruiting body, developmental stages, age of the mycelium, biochemical composition, interval between fructifications, and element accumulation in mushrooms is also influenced by environmental factors, such as the amount of organic matter, pH, temperature, water content, and element concentrations in the soil [9,11,12]. In urban areas, soils are additionally burdened by the influence of traffic and industrial pollution, which may lead to the accumulation of heavy metals and metalloids that are of particular importance for human health due to their toxic effects even at low concentrations, such as As, Cd, and Pb [3,13,14,15]. Mushrooms growing near pollution sources are generally avoided for consumption, and those from forests are considered safe, while the geochemical composition of the underlying soil is usually neglected as a potentially significant source of elements. This is particularly important when considering mushrooms growing in areas with naturally elevated concentrations of metals in the soil [12]. Consequently, elevated levels of metals and metalloids in mushrooms have been reported not only near industrial areas [16,17] and intensive traffic [9,10,13,16,18,19], but also in areas with naturally elevated metal concentrations due to the geological composition of the bedrock [12].

To date, numerous studies have been carried out on the content of individual elements in fruiting bodies from different areas [20], such as France [21], the Czech Republic [22], Poland [4,23,24,25], Slovakia [6], Spain [9,10,26], Portugal [16], Turkey [27,28,29,30], USA [31], China [32,33], Greece [18], Serbia [34,35], and Italy [36,37,38,39]. However, published results often differ significantly and usually include a narrow range of elements and/or mushroom species studied [20]. Studies on the metal content in mushrooms from Croatia are also sparse and mostly limited to several measured elements [40,41,42].

In this work, we report on the concentration of 46 elements, including macroelements, trace elements, and rare earth elements with Y (REY), determined in 15 edible wild mushroom species from urban and forest areas in northwestern Croatia. As this region is characterized by naturally elevated levels of aluminum (Al), bismuth (Bi), cobalt (Co), chromium (Cr), cesium (Cs), iron (Fe), molybdenum (Mo), nickel (Ni), lead (Pb), antimony (Sb), scandium (Sc), titanium (Ti), thallium (Tl), uranium (U), vanadium (V), zinc (Zn), and rare earth elements, including yttrium (REY) [12,43,44], the distribution of elements in edible mushroom species from the study area will provide new insights into metal uptake by mushroom fruiting bodies.

This comprehensive study aims to increase the knowledge on element distribution in different edible mushrooms, and particularly to investigate the importance of site-specific properties (soil geochemistry) versus the species-specific uptake of elements in the investigated mushrooms and the influence of urban pollution on element uptake by mushrooms. This was accomplished by (i) studying the distribution of elements in different mushrooms from specific locations, and (ii) comparing element content in mushrooms from urban and forest locations. To assess the health risks associated with the consumption of the wild mushrooms studied, the daily intake and hazard index for elements that may have a negative impact on human health were also calculated.

## 2. Materials and Methods

### 2.1. Study Area

**Figure 1:**
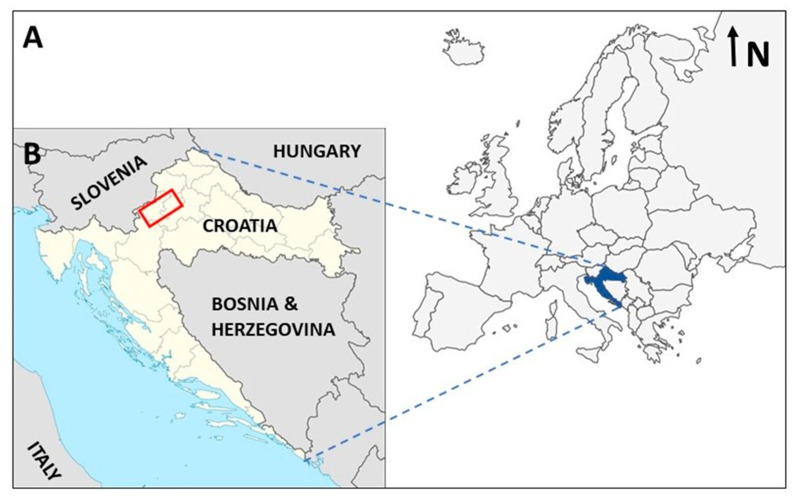
Map of the study area, its geographical position (**A**), and sampling area (**B**).

During the sampling campaign in October 2016, an attempt was made to collect different edible wild mushroom species from the urban and forest areas (Table 1); mushrooms of a particular species were collected from different locations, and different species were collected from each sampling location. Sampling was conducted in northwestern Croatia (Figure 1), in green areas within the capital city of Zagreb, and in rural parts of Karlovac County. The city of Zagreb has a population of approximately 800,000 people inhabiting an area of 641 km^2^. Green areas of the city where the mushrooms were collected include forested areas of the city parks: Dotrščina (2 km^2^; samples 1/7272–1/7275), Jelenovac (54 ha; samples 1/7258–1/7263), and Maksimir (316 ha; samples 1/7267–1/7269), as well as smaller green areas in residential areas of the city (samples 1/7256, 1/7293, and 1/7294). These mushrooms, although exposed to urban pollution due to their location in the capital, were not sampled in the immediate vicinity of roads, but in the central areas of city parks. Only samples 1/7293 and 1/7294 were collected in the immediate vicinity of a road with low traffic volume. The city of Zagreb, despite being a capital city with industries and dense traffic, had good air quality with low PM10 content at the time of sampling, as shown by the data collected by the Croatian Environmental Agency. The samples from Karlovac County (1/7284, 1/7288, 1/7291, and 1/7292) included various forest sites distributed throughout the district, far from roads and other pollution sources.

A total of 19 samples, representing 15 edible mushroom species from the phylum Basidiomycota, were collected and analyzed for total element concentration (Table 1). Photographs of the identified mushroom species and details of collection, morphological and molecular identification, and screening of bioactive compounds can be found elsewhere [45]. Basic information on the mushroom species and sampling is also presented here (Table 1) to be easily accessible for interpretation of the data from this study. All mushrooms used in this study are stored in the Croatian National Fungarium (CNF).

### 2.2. Sample Collection and Preparation

In the laboratory, soil particles were carefully removed from the fruiting bodies. Approximately 5 g of raw fruiting bodies from each sample was stored in a separate plastic bag at −82 °C. Prior to analyses, the frozen samples were freeze-dried (Finn-Aqua Lyovac GT 2) and pulverized with liquid nitrogen.

### 2.3. Multi-Element Analysis

Prior to multi-element analysis, subsamples (0.05 g) of the previously freeze-dried samples were subjected to digestion in the microwave oven (Multiwave ECO, Anton Paar, Graz, Austria) with 7 mL of HNO_3_ (65%, *supra pur*) and 0.1 mL of HF (48%, *pro analysi*) [46]. Digests were acidified with 2% (*v/v*) HNO_3_ (65%, *supra pur*), without further dilution, and indium (In, 1 µg L^−1^) was added as an internal standard.

The multi-element analysis was performed by High-Resolution Inductively Coupled Plasma Mass Spectrometry (HR-ICP-MS) using an Element 2 instrument (Thermo, Bremen, Germany). The instrument conditions and measurement parameters used in this work are described in the ref. [47]. Standards for multi-element analysis were prepared by appropriate dilution of a multi-element reference standard (Analytika, Prague, Czech Republic) containing Al, As, Be, Cd, Co, Cr, Cu, Fe, Li, Mn, Ni, Pb, Rb, Sr, and Ti, to which single-element standard solutions of Sn (Analytika, Prague, Czech Republic) and Sb (Analytika, Prague, Czech Republic) were added. For the analysis of REY, separate standards were prepared by appropriate dilution of a multi-elemental reference standard (Analytika, Prague, Czech Republic) containing Ce, Dy, Er, Eu, Gd, Ho, La, Lu, Nd, Pr, Sm, Tb, Tm, Y, and Yb.

The samples were analyzed for the total concentration of 46 elements (Al, As, Ba, Be, Bi, Ca, Cd, Ce, Co, Cr, Cs, Cu, Dy, Er, Eu, Fe, Gd, Ho, K, La, Li, Lu, Mg, Mn, Mo, Na, Nd, Ni, Pb, Pr, Rb, Sb, Sc, Se, Sn, Sm, Sr, Tb, Ti, Tl, Tm, U, V, Y, Yb, and Zn). All concentrations refer to dry matter.

### 2.4. Quality Control

Limits of detection (LOD) and limits of quantification (LOQ) were calculated as 3 and 10 times, respectively, the standard deviation of 10 consecutive measurements of analyte concentration in the procedural blank. Quality control of the analytical procedure was performed by simultaneous analysis of the blank sample and the certified reference material for Citrus leave (NCS ZC73018, China National Analysis Center for Iron and Steel). Good agreement was obtained between the analyzed and certified concentrations for all the elements measured, with recoveries ranging from 85% to 106%. Measurement precision was determined from five consecutive measurements in two Citrus leave CRM samples, and averaged 5%.

### 2.5. Health Risk Assessment

The assessment of non-carcinogenic health risks of heavy metals from mushroom consumption was performed according to the USEPA procedure described in the ref. [48]. The risk assessment included metals known to be harmful to human health, such as As, Ba, Be, Cd, Cu, Fe, Mn, Ni, Pb, Sb, Sr, You, and Zn. First, the intake factor, that is, the estimated daily intake, was calculated according to Equation (1):

(1) $$EDI\;\left({µg\;\mathrm{kg}}^{-1}{\;\mathrm{day}}^{-1}\right)=\frac{C_{metal\;}\times \;\mathrm{IR}\;\times \;\mathrm{ED}\;\times \;\mathrm{EF}}{\mathrm{BW}\;\times \;\mathrm{AT}}\;,$$

where *C_metal_* is the metal concentration in dried mushrooms (µg kg^−1^), IR is the intake rate (in kg per person per day) corresponding to 0.03 kg of dried mushrooms [11], ED is the exposure duration (30 years for adults), EF is the exposure frequency (350 days per year^−1^), BW is the average body weight (70 kg for adults), and AT is the average exposure duration (10,500 days, calculated *C_meta_* as EF × ED).

Then, the target hazard quotient (*THQ*) of a given metal (Equation (2)) was calculated as the ratio between the estimated daily intake (*EDI*) and the reference dose (*RfD*). The *RfD* value is an estimate of the daily dose that is unlikely to pose a significant risk to human health over a lifetime [49]. This value is specific to the element being assessed and is defined in Integrated Risk Information System assessments (IRIS) under the USEPA programme.

(2) $$THQ=\frac{EDI\;\left({µg\;\mathrm{kg}}^{-1\;}{\mathrm{day}}^{-1\;}\right)}{RfD\;\left({µg\;\mathrm{kg}}^{-1\;}{\mathrm{day}}^{-1\;}\right)}\;$$

Finally, the risk assessment related to the cumulative effect of several heavy metals present in the mushrooms was performed by calculating the overall hazard index as the sum of exposures scaled by the toxicity of each metal (Equation (3)),

(3) $$HI=\overset{n}{\underset{i=1}{\sum }}THQ_{i}\;,$$

where *THQ_i_* is the target hazard quotient for a single metal.

### 2.6. Statistical Analysis

Data were statistically analyzed using STATISTICA 7.0 (StatSoft Inc., Tulsa, OK, USA). The Spearman rank correlation coefficient was used for correlation of investigated elements in studied mushrooms. Multivariate principal component analysis (PCA) was performed on the data matrix consisting of element concentrations. The significance level was set at *p* < 0.05.

## 3. Results

The concentrations of macro- and trace elements determined in the studied mushrooms, along with calculated LODs and LOQs, are presented in Table 2. Elements that showed the widest range of concentrations among different mushrooms were As, Co, Cs, Mo, Rb, and Tl, while Be, Cr, Fe, K, Li, Mg, Mn, Ni, Sn, and Zn were present in a narrow range. The measured elements appeared in the following order of abundance: K > Mg > Na > Ca > Rb > Zn > Fe > Al > Cu > Mn > Ti > Cd > Pb > As > Cs > Ba > Ni > Se > Sr > Mo > ∑REY > Cr > Co > V > Sn > Tl > Li > Sb > U > Bi > Be.

The results of the measurement of REY (La, Ce, Pr, Nd, Sm, Eu, Gd, Tb, Dy, Ho, Er, Tm, Yb, Lu, and Y), along with calculated LODs and LOQs, are shown in Table 3. The description of REY distribution in the samples was based on geochemical classification into light (LREE—La, Ce, Pr, Nd, Sm, Eu, Gd) and heavy (HREE—Tb, Dy, Ho, Er, Tm, Yb, Lu, and Y). The concentrations of REY in all the analyzed samples ranged over three orders of magnitude, from below the detection limit (Eu, Gd, Tb, Ho, Er, Tm, and Lu in some samples) to 0.137 mg kg^−1^ (Ce), with ΣREY ranging from 0.031 mg kg^−1^ to 0.452 mg kg^−1^ (Table 3). Among them, Ce was present in the highest concentrations in all samples and accounted for between 19% and 36% of the total REY, while Eu, Gd, Tb, Ho, Tm, and Lu had the lowest values. In general, the mushrooms showed great variability in terms of REY concentrations, with an RSD of up to 120% and ΣREY variability of up to one order of magnitude. The highest REY values were measured in *L. deterrimus* (1/7284, 0.452 mg kg^−1^), while the lowest were observed in *P. dryinus* (1/7256, 0.031 mg kg^−1^). LREEs were found to be more abundant in the majority of the samples studied, with LREE/HREE ratios ranging from 0.7 to 3.8 and the average being 1.9. The content of LREEs accounted for 41.9–79.3% of the total REYs in the mushrooms studied.

## 4. Discussion

### 4.1. Toxic Elements (Pb, Cd, As) 

The non-essential metals Pb, Cd, and As show adverse effects on human health even at low concentrations. They readily accumulate in living organisms and interfere with metabolic processes and biological functions. In the investigated mushrooms, with a few exceptions, they were mostly present in low concentrations. A comparison of measured element concentrations with those from other studies in the region is shown in Table 4.

The content of lead in the studied mushrooms ranged from 0.072 mg kg^−1^ in *P. dryinus* (1/7256) to 1.11 mg kg^−1^ in *L. excipuliforme* (1/7259) (Table 2). The exception was *C. cornucopioides* (1/7292) with the determined concentration of 4.02 mg kg^−1^. These values are within the usual range of concentrations of Pb in wild mushrooms from uncontaminated sites (<5 mg kg^−1^; [52]). Pb concentrations in certain species (*A. cylindracea*, *M. procera*, *L. deterrimus*, *C. comatus*, *L. nuda*, *L. leucothites*, and *L. perlatum*) were sometimes significantly lower compared to available data from other studies in the region [35,40,41,42] (Table 4) and in other areas [10,19,40,53,54,55].

Uptake from contaminated soil, where it is mostly deposited by atmospheric processes, is considered the main source of Pb in wild mushrooms [19]. Although its use in gasoline has been banned, traffic is still the main source of Pb in urban soils due to its accumulated amounts [15], and recent studies have demonstrated a direct influence of traffic on Pb concentrations [16,19].

The narrow range of Pb concentrations in this study suggests a limited influence of traffic on Pb content in the studied mushrooms. This is also supported by the low content in the species *C. comatus* (1/7274), *L. perlatum* (1/7260, 1/7267), and *L. nuda* (1/7291), which readily accumulate Pb and are considered good indicators of traffic pollution [9,52]. However, it should be noted that mushrooms were mostly sampled from larger green areas within the city, while in studies where traffic pollution caused elevated Pb levels in mushrooms, samples were collected closer to roads (up to 50 m), which may have contributed to pollution levels [9,13,16,56].

Schlecht and Säumel [19] found that although traffic pollution undoubtedly affected Pb levels in mushrooms, high Pb concentrations were also determined in mushrooms from areas where low urban pollution is expected, while low concentrations were found in mushrooms from potentially polluted areas [17]. Although the Pb concentrations determined in this study do not exceed the usual level in mushrooms, it can be observed that the highest concentration was determined in *C. cornucopioides* (1/7292) sampled in a forest far from any significant source of pollution, such as the impact of the industry or traffic (Table 2), implying that Pb uptake in this case is mainly determined by the geochemistry of the underlying soil. This is supported by the fact that this species also had elevated or maximal concentrations of lithogenic elements (Al, Be, Cr, Cs, Li, Ni, Rb, REY, Ti, and V; Table 2). However, genetic factors appeared to predominate in Pb uptake by *L. deterrimus*, as similar Pb concentrations were obtained in the two samples from distant sites (1/7284 and 1/7288).

Cadmium concentrations in the investigated mushrooms varied from 0.116 mg kg^−1^ in *H. radicata* (1/7275) to 0.681 mg kg^−1^ in *P. dryinus* (1/7256), with the exception of *L. leucothites* (1/7293) and *A. cylindracea* (1/7294), for which much higher values (4.55 mg kg^−1^ and 3.96 mg kg^−1^, respectively) were obtained (Table 2). The values obtained are in agreement with previous studies from the region [40,41,42,50,51] (Table 4) and are within the accepted range of values for wild mushrooms (0.5–5 mg kg^−1^; [52]). The values obtained for certain species (*C. comatus*, *L. nuda*, *L. perlatum*, *M. procera*) are similar to those previously determined [40,53]. However, higher [33,54,57,58], as well as lower [27,53,55] concentrations have also been reported.

According to Melgar et al. [58], bioaccumulation of Cd by mushrooms is species-dependent, and the age of the mycelium and the period between fructifications are suggested as important factors in determining the metal content. However, higher Cd levels in plants, like Pb, are mostly associated with urban contamination [59]. Schlecht and Säumel [19] found that traffic pollution affected Cd levels only in some mushroom species, highlighting site-specific characteristics as a determining factor for uptake. This is supported by the results of our study, as the two mushroom species (*L. leucothites* (1/7293) and *A. cylindracea* (1/7294)) with higher Cd levels (Table 2) were sampled at the same site, near a road in the capital city of Zagreb (Table 1), and thus were exposed to the influence of nearby traffic. Considering that these two species have different lifestyles, that is, grow on different substrates (*L. leucothites* in soil and *A. cylindracea* on wood), but both have significantly elevated Cd levels, it seems that the sampling location, such as soil properties and/or urban pollution, had a predominant influence on the uptake of Cd by these mushrooms. A positive correlation between Cd and typical lithophilic elements, such as Cs and Rb (r = 0.53 and 0.51, respectively, *p* < 0.05; Table S1), confirms that soil geochemistry is an important factor in Cd uptake.

Arsenic concentrations were below 0.7 mg kg^−1^ (Table 2), with the exception of *P. multipedata* (1/7263), in which 3.68 mg kg^−1^ was determined. Other mushrooms from the same sampling area (Jelenovac; 1/7258, 1/7259, 1/7260, 1/7262) or the same family (*Psathyrellaceae*; 1/7268 and 1/7273) had significantly lower values, indicating species-specific affinities as the main source of the elevated As content. However, *P. multipedata* was not recognized as an As accumulator in previous studies, and this maximum is outside the accepted range for As concentration for mushrooms from uncontaminated areas (<1 mg kg^−1^, [52]).

Vetter [60] found that the level of As in a given species is the result of several factors, both environmental and genetic, with some species having an accumulative affinity for As, regardless of their habitat. In this study, lower As levels were found in mushrooms growing on wood (1/7256, 1/7268, 1/7273, 1/7275, 1/7294), supporting the work of Falandysz and Rizal [61], who propose geogenic sources as the most important factor for As uptake.

The established maximum level for Cd and Pb in edible mushrooms is regulated by EU regulations [62,63] and is 0.3 mg kg^−1^ for Pb and 1 mg kg^−1^ for Cd, wet weight (3 and 10 mg kg^−1^ dry weight at 90% moisture content). The maximum amount of As in fresh mushrooms is regulated by Croatian legislation to 0.3 mg kg^−1^ (3 mg kg^−1^ dry weight) [64]. According to these regulations, *P. multipedata* (1/7263) and *C. cornucopioides* (1/7292) collected in the forest area far from direct anthropogenic influences are not considered safe for consumption due to elevated As and Pb content, respectively. Cd levels were well below the maximum allowable limit in dry products. These results support the conclusions of Fu et al. [32] who advise caution in the consumption of wild mushrooms due to possible accumulation of these potentially toxic elements.

### 4.2. Macronutrients (Ca, Mg, K, Na)

In general, the highest concentrations of macroelements were determined in mushrooms belonging to the *Psathyrellaceae* family, while *C. comatus* (1/7274) was rich in Ca and Na.

The determined concentrations of Ca, Mg, and K were in the usual range for wild mushrooms (100–500 mg kg^−1^, 800–1800 mg kg^−1^, and 20–40 g kg^−1^, respectively; [50]), although the values for individual mushroom species (*L. perlatum*, *M. procera*, *C. cornucopioides*, *L. nuda*, *A. cylindracea*, *C. comatus*) did not always agree with those determined by other researchers [23,52,53,56].

Concentrations of Ca ranged from 128 to 545 mg kg^−1^ (Table 2), except in *P. dryinus* (1/7256), where an exceptionally low concentration (26 mg kg^−1^) was determined. The concentrations of Mg and K varied from 0.939 to 1.88 g kg^−1^ and 17.1 to 49.5 g kg^−1^, respectively, except for mushrooms from the family *Psathyrellaceae* (*P. piluliformis* (1/7268, 1/7273) and *P. multipedata* (1/7263)), where slightly higher values of Mg (around 2 g kg^−1^) and significantly higher values of K (65.3–80.8 g kg^−1^) were determined. The determined Na concentrations were outside the range suggested by Kalač [52] (100–400 mg kg^−1^); in the majority of samples they were below 100 mg kg^−1^, while in mushrooms from the family *Psathyrellaceae* (*P. piluliformis* (1/7268, 1/7273) and *P. multipedata* (1/7263)) and *L. leucothites* (1/7293) and *C. comatus* (1/7274) from the family *Agaricaeae* were in the range of 0.653 to 1.01 g kg^−1^ (Table 2).

While the uptake of K was correlated with that of Mg and Na (r = 0.6 and 0.7, respectively, *p* < 0.05; Table S1), taking into account all studied mushrooms, the uptake of Na was significantly increased in species of the family *Psathyrellaceae* from different sites, but was also site-specific, as samples 1/7273, 1/7274, and 1/7275 from Dotrščina Park had significantly increased values compared to other mushrooms. However, the fact that mushrooms of the same species collected from different sites (*L. perlatum* (1/7260 and 1/7267), *I. gibba* (1/7258 and 1/7272) and *P. piluliformis* (1/7268 and 1/7273)) had similar concentrations of macroelements supports the finding that macronutrient uptake is mostly species-dependent. *L. deterrimus* (1/7284 and1/7288), a mycorrhizal mushroom, showed different concentrations of K and Na, which could indicate a possible importance of the individual host tree in the uptake of major elements.

### 4.3. Micronutrients (Cu, Fe, Mn, Mo, Se, Zn)

The obtained values for micronutrients were within the accepted range for wild mushrooms (20–100 mg kg^−1^ for Cu, 30–150 mg kg^−1^ for Fe, 10–60 mg kg^−1^ for Mn, <2 mg kg^−1^ for Se, 25–200 mg kg^−1^ for Zn; [52]). Only for Mo, several samples exceeded the values found in Kalač [52] (<0.6 mg kg^−1^).

The concentrations of Cu and Fe ranged from 2.19 to 84.3 mg kg^−1^, and 11.0 to 105 mg kg^−1^, respectively (Table 2). The lowest values were found in *P. dryinus* (1/7256), and the highest in *L. perlatum* (1/7260), and no site-, species-, or lifestyle-related trends were observed. A comparison with data from related studies for specific mushrooms (*L. perlatum*, *C. comatus*, *M. procera*, *L. nuda*, *C. cornucopioides*, *L. leucothites*, *L. deterrimus*, *A. cylindracea*) revealed similar, but also divergent values [23,40,53,54,55,56,57,65].

The values for Mn ranged from 4.9 mg kg^−1^ to 38.2 mg kg^−1^, with the lowest concentrations obtained in *P. dryinus* (1/7256), *L. deterrimus* (1/7288), and *A. cylindracea* (1/7294), and the highest in *L. perlatum* (1/7267), *I. gibba* (1/7272), and *L. nuda* (1/7291). In general, the values obtained for certain species (*L. perlatum*, *M. procera*, *C. comatus*, *L. nuda*, *C. cornucopioides*) were lower than those reported in the literature [23,28,33,57,65], although higher values were found for *L. nuda* [29] and similar values for *A. cylindracea* and *C. comatus* [53], *L. leucothites* [53], and *M. procera* [23,30].

A positive correlation between Mn and Fe (r = 0.62, *p* < 0.05) and Ni and Fe (r = 0.53, *p* < 0.05; Table S1) in the investigated mushrooms suggests their interdependent uptake, as previously reported by Kokkoris et al. [18]. The co-occurrence of Mn and Fe results from the Fe-Mn oxides and oxyhydroxides associated with clay minerals in the soil. Moreover, a strong correlation was observed between V and Fe (r = 0.66, *p* < 0.05; Table S1) due to its association with Fe oxides [3]. This agrees with previously reported findings that Fe, Mn, and partially Ni in topsoils from the Zagreb area are mostly of geological/pedological origin [66].

The determined concentrations of Mo ranged from 0.007 mg kg^−1^ to 1.15 mg kg^−1^. The lowest Mo content was determined in *L. deterrimus* (1/7284, 1/7288), *P. dryinus* (1/7256), and *P. piluliformis* (1/7268, 1/7273), while *P. flaccida* (1/7262) and *L. nuda* (1/7291) exceeded the usual content for mushrooms from uncontaminated areas [52]. In general, it was observed that mushrooms growing on wood had lower Mo concentrations.

Selenium is a micronutrient normally present in mushrooms at 2 mg kg^−1^ [52]. This is in agreement with the results obtained; the concentrations obtained were mostly <1 mg kg^−1^, except for the two samples with higher values; 1.59 mg kg^−1^ in *P. multipedata* (1/7263) and 1.49 mg kg^−1^ in *L. leucothites* (1/7293). The important role of Se in biological functions is supported by its positive correlation with macronutrients K and Mg (r = 0.62 and 0.5, respectively; *p* < 0.05; Table S1).

Concentrations of Zn ranged from 17.3 mg kg^−1^ in *P. dryinus* (1/7256) to 143 mg kg^−1^ in *L. excipuliforme* (1/7259). *L. perlatum* (1/7260 and 1/7267), as an accumulator of Zn [52], showed higher values; however, they were not the highest recorded. Species from the *Psathyrellaceae* family (*P. multipedata* (1/7263) and *P. piluliformis* (1/7268 and 1/7273)) and Russulaceae (*L. deterrimus*, 1/7284 and 1/7288), which were from different sites, showed similar Zn values, suggesting that species-specific affinities rather than soil geochemistry are important in Zn uptake. Considering specific mushroom species, similar values were previously reported for *C. comatus* [27,30], *M. procera* [23,30,40], *L. leucothites* [67], *L. deterrimus* [40], and *A. cylindracea* [53], but different concentrations can also be found [33,53,55,57,65].

### 4.4. Lithogenic Group of Elements 

The group of lithogenic elements (Al, Ba, Li, Ti, Cs, and Rb) was positively correlated with each other (r = 0.50–0.87, *p* < 0.05; Table S1), indicating a common mechanism of their uptake. Their concentrations were in a narrow range of values, considering the differences between localities. Positive correlations of REY, Sr, and V with Al (Table S1) indicate their common origin from the soil.

Concentrations of Al varied from 7.83 mg kg^−1^ in *P. dryinus* (1/7256) to 99.3 mg kg^−1^ in *P. piluliformis* (1/7268). Similar values were reported for *C. comatus* [53] and *L. nuda* [28], lower for *A. cylindracea* [53] and *C. cornucopioides* [28], and higher for *L. perlatum* [28]. According to the data for a number of wild species [68], the Ba content in mushrooms is mostly below 1.6 mg kg^−1^. This is in agreement with the present results; only *P. piluliformis* (1/7268) slightly exceeded this range with a value of 1.73 mg kg^−1^. Similar concentrations of Ba were previously reported for *C. comatus*, and higher for *A. cylindracea* [53]. Lithium is not a commonly occurring element in mushrooms, and the results obtained are consistent with the usual reported range, such as below 0.19 mg kg^−1^ [69]. The content of Ti was up to 9.7 mg kg^−1^, in agreement with the usual content in wild mushrooms (<10 mg kg^−1^, [52]), although Niedzielski et al. [53] found lower content in *A. cylindracea* and *C. comatus*. The Cs content was mostly below 0.6 mg kg^−1^, except in *C. cornucopioides* (1/7292), where the determined value was 1.96 mg kg^−1^, confirming an increased input of lithogenic elements, as previously suggested. A strong positive correlation between Cs and Rb (r = 0.85, *p* < 0.05; Table S1) results from their similar geochemical behaviour [70]. According to data collected by Kalač [52], Rb is present in mushrooms in tens to hundreds of mg kg^−1^; in this study, the content of Rb varied from 2.59 mg kg^−1^ in *L. perlatum* (1/7260) to 299 mg kg^−1^ in *C. cornucopioides* (1/7292).

The highest concentrations of Cr and Ni were determined to be 0.761 mg kg^−1^ and 1.595 mg kg^−1^, respectively, in *C. cornucopioides* (1/7292) (Table 2). The concentrations of Ni and Cr were generally lower compared to other studies [33,41,42,55], while similar levels of Ni were determined in *A. cylindracea* [53]. The results showed equal Ni content in the two samples of *L. deterrimus* sampled at widely separated sites (1/7284 and 1/7288), while similar values were also observed in mushrooms belonging to the *Tricholomataceae* family (1/7258, 1/7262, 1/7272, 1/7291) (Table 2); this may indicate that the accumulation of Ni is species-specific. However, the positive correlation of Ni with Fe and V (r = 0.53, *p* < 0.05; Table S1) suggests its association with Fe oxides and oxyhydroxides from soil. In contrast, the lack of a significant correlation between Cr and other elements suggests that there are multiple factors controlling its bioaccumulation.

### 4.5. Rare Earth Elements 

The REY concentrations obtained (Table 3) are in accordance with the values reported for saprobic macrofungi [71] and aboveground species [72], but are significantly higher compared to the values reported for *Suillus luteus* [70] and lower compared to the values reported by the ref. [73] for caps of *M. procera* (Table 5). Moreover, the mushrooms from northwestern Croatia investigated in this study have much lower REY values compared to the mushrooms of the genus *Agaricus* from the Prašnik area (eastern part of Croatia; [44]), although all of them were grown on a pedological substrate enriched with REEs [43]. The reason for the observed discrepancy is probably the fact that mushrooms of the genus *Agaricus* are known to easily accumulate metals [11].

In general, the results indicate a large variability between the different species studied in terms of their ability for REY uptake and suggest that both the soil substrate and the mushroom species influence the accumulation of this group of elements in the mushroom tissue.

### 4.6. Factors Influencing the Element Uptake 

**Figure 2:**
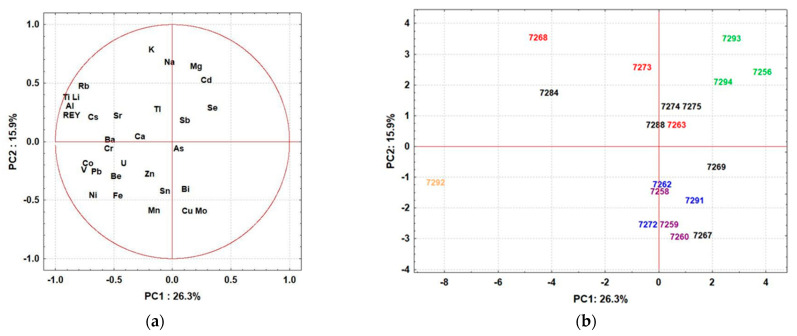
Principal component analysis: PC1–PC2 loading (**a**) and score plot (**b**).

In order to identify the role of different factors affecting the intake of elements, such as species, lifestyle, dietary habits, and geographical location, the relationship between the obtained element concentrations was investigated using principal component analysis (PCA). The eigenvalues of the first two principal components (PCs) were greater than 1, indicating their significance. The first two PCs explained 42.2% of the total variability among 32 variables; the first component (PC1) contributed 26.3%, while the second corresponded to 15.9% of the total variance of the data set. PCA results are presented on the PCA loading plot (Figure 2a) and PCA score plot (Figure 2b), which illustrate the alignment of variables (elements) and samples (mushrooms) with respect to principal components.

Based on these parameters, the following can be observed:

(i) Mushrooms belonging to the *Psathyrellaceae* family (1/7263, 1/7268, and 1/7273; colored red in Figure 2b) showed high variability in element distribution, suggesting that soil geochemistry/properties are the main factor determining the uptake of elements. However, *P. piluliformis* (1/7268 and 1/7273) collected from widely separated sites showed similarities in terms of high uptake of macronutrients (K, Na, Mg) and low uptake of Mo and Cu, indicating that the uptake of these elements is species-specific.
(ii) *L. leucothites* (1/7293) and *A. cylindracea* (1/7294) sampled from the same site in the city of Zagreb, and *P. dryinus* (1/7256) (all colored green in Figure 2b) from another site within the urban area were characterized by high Cd concentrations; these mushrooms were collected near a road and, therefore, the elevated Cd levels could be explained by traffic pollution. However, elevated levels of other elements derived from traffic pollution, such as Pb, were not observed.
(iii) Mushrooms from the *Tricholomataceae* family (1/7262, 1/7272, and 1/7291; colored blue) were grouped independently of sampling sites, suggesting that species-specific affinities in element uptake predominate. These mushrooms were described by slightly higher levels of Cu, Mn, and Mo and lower levels of Cd and macronutrients.
(iv) Significant similarity in the content of elements was observed in different mushrooms sampled within the green area at the Jelenovac site: *L. excipuliforme* (1/7259), *I. gibba* (1/7258), *L. perlatum* (1/7260) (all colored purple in Figure 2b) and *P. flaccida* (1/7262) (colored blue in Figure 2b). This strongly suggests that geochemistry and soil properties are the main factors controlling elemental uptake, as these samples are characterized by higher levels of Cu, Mn, and Mo and lower levels of K, Mg, and Na.
(v) PCA analysis confirmed the unique elemental composition of *C. cornucopioides* (1/7292; colored orange in Figure 2b), which is distinguished from all other samples by its significantly elevated values of Co, Ni, Pb, and REY.

The results presented showed that mushrooms belonging to the same species or family collected in different areas had considerably different elemental contents, suggesting different soil geochemical composition and/or anthropogenic influences as the main factors determining elemental content. Since the main distribution of trace elements in the studied areas did not indicate an anthropogenic influence, it can be assumed that soil geochemical properties are the most important factor determining these variations in chemical composition. The influence of different soil properties and geochemistry on the uptake of elements by mushrooms may be more evident in studies comparing soils with markedly different geological composition, as in the study by Nikkarinen and Mertanen [74] and Alaimo et al. [37], than when comparing mushrooms growing in areas where differences in soil geochemistry are less pronounced. However, several elements, such as As, Ni, Zn, and macronutrients, showed similar behavior in certain mushroom species or families, indicating an important influence of species-specific affinity for certain elements. Finally, both factors—soil geochemistry and species characteristics—have an important influence on the uptake of elements, as also shown by other studies [36,38,39,52], although the prevalence of each factor varies from species to species and depends on the element in question.

### 4.7. Health Risk Assessment 

The data (THQ and HI values) obtained in the assessment of non-carcinogenic health risk due to metal ingestion from the consumption of the wild mushrooms studied are presented in Table 6. Toxicity was evaluated based on the calculated HI values. If the HI value for a mixture is less than 1, it is considered unlikely that assessed exposure from mushroom consumption will result in significant toxicity. On the other hand, there is great concern about potential toxicity when the HI value is greater than 1 [48].

The risk assessment revealed that 14 of the 19 mushrooms studied, from both urban and forest areas, had HI levels above 1; therefore, consumers of these mushrooms are exposed to a significant non-carcinogenic health risk due to the high metal content. As, Cd, and Cu, which have the highest THQ values, account for the largest proportion of total toxicity.

The mushrooms that do not pose a risk to human health are those growing on wood—1/7256, 1/7268, 1/7273, and 1/7275. The only mushroom growing in soil that is considered safe for consumption is *L. deterrimus* (1/7288), which was growing in grassland within the forest area.

Therefore, a diet that includes many wild mushrooms can be very risky. Dowlati et al. [48] conducted a comprehensive review, meta-analysis, and evaluation of non-carcinogenic health risk to humans based on the collected data on the concentrations of toxic metals in edible mushrooms from different countries of the world. The health risk assessment revealed that consumers from 8 out of 19 countries studied were exposed to a significant non-carcinogenic health risk.

## 5. Conclusions

In the studied mushrooms, the most abundant elements were essential nutrients, whose uptake seems to be more under a genetic influence and less influenced by site-specific characteristics.

The content of toxic elements was low, and no influence of traffic pollution could be detected, as similar concentrations of Pb were found in mushrooms from forest and urban sites. While the concentrations of metals did not show variations according to substrate (wood and soil), it was observed that mushrooms growing on wood generally had lower levels of As and Mo.

Nonetheless, the health risk assessment revealed a high non-carcinogenic risk of metals in 14 out of 19 mushrooms studied from both urban and forest areas, suggesting that these mushrooms should be consumed with caution.

Despite a limited data set, the results suggest that both the geochemical composition of substrate and the species-specific affinities influence the uptake of elements, with the predominance of one of these factors depending on both the mushroom species and the element of interest. The diversity of factors affecting the chemical composition of edible mushrooms should certainly be considered not only in future studies, but also in the evaluation of edible mushrooms as a source of certain metals in the diet.

## Figures and Tables

**Table 1:** Investigated species of mushrooms, and their lifestyle and location information. Trophic mode (**^a^** saprotroph, ^b^ pathotroph/saprotroph or **^c^** symbiotroph).

	Taxon/Family	Locality	Habitat	Substrate
*Urban area, Zagreb*				
1/7256 ^b^	*Pleurotus dryinus* *Pleurotaceae*	Črnomerec	forest of *Populus nigra, Acer campestre,* *Sambucus nigra*	wood
1/7258 ^a^	*Infundibulicybe gibba* *Tricholomataceae*	Jelenovac	forest of *Quercus robur, Carpinus betulus,* *Fagus sylvatica*	soil
1/7259 ^a^	*Lycoperdon excipuliforme* *Agaricaceae*	Jelenovac	forest of *Quercus robur, Carpinus betulus,* *Fagus sylvatica*	soil
1/7260 ^a^	*Lycoperdon perlatum* *Agaricaceae*	Jelenovac	forest of *Quercus robur, Carpinus betulus,* *Fagus sylvatica*	soil
1/7262 ^a^	*Paralepista flaccida* *Tricholomataceae*	Jelenovac	forest of *Quercus robur, Carpinus betulus,* *Fagus sylvatica*	soil
1/7263 ^a^	*Psathyrella multipedata* *Psathyrellaceae*	Jelenovac	forest of *Quercus robur, Carpinus betulus,* *Fagus sylvatica*	soil
1/7267 ^a^	*Lycoperdon perlatum* *Agaricaceae*	Maksimir Park	forest of *Quercus robur, Q. petraea,* *Carpinus betulus*	soil
1/7268 ^a^	*Psathyrella piluliformis* *Psathyrellaceae*	Maksimir Park	forest of *Quercus robur, Q. petraea,* *Carpinus betulus*	wood
1/7269 ^a^	*Macrolepiota procera* *Agaricaceae*	Maksimir Park	forest of *Quercus robur, Q. petraea,* *Carpinus betulus*	soil
1/7272 ^a^	*Infundibulicybe gibba* *Tricholomataceae*	Dotršćina Park	forest of *Quercus petraea,* *Carpinus betulus, Fagus sylvatica*	soil
1/7273 ^a^	*Psathyrella piluliformis* *Psathyrellaceae*	Dotršćina Park	forest of *Quercus* sp., *Carpinus betulus*	wood
1/7274 ^a^	*Coprinus comatus* *Agaricaceae*	Dotršćina Park	forest of *Quercus* sp., *Carpinus betulus*	soil
1/7275 ^a^	*Hymenopellis radicata* *Physalacriaceae*	Dotršćina Park	forest of *Quercus* sp., *Carpinus betulus*	wood
1/7293 ^a^	*Leucoagaricus leucothites* *Agaricaceae*	Ruđer Bošković Institute	grassland	soil
1/7294 ^b^	*Agrocybe cylindracea* *Strophariaceae*	Ruđer Bošković Institute	grassland, on old living tree of *Populus nigra*	wood
*Forest area, Karlovac County*				
1/7284 ^c^	*Lactarius deterrimus* *Russulaceae*	Dvorišće Ozaljsko	grassland, near planted *Picea abies*	soil
1/7288 ^c^	*Lactarius deterrimus* *Russulaceae*	Vukova Gorica	grassland, near planted *Picea abies*	soil
1/7291 ^a^	*Lepista nuda* *Tricholomataceae*	Vukova Gorica	forest of *Quercus robur, Corylus avellana*	soil
1/7292 ^c^	*Craterellus cornucopioides* *Cantharellaceae*	Novaki Ozaljski	forest of *Fagus sylvatica*	soil

**Table 2:** Concentrations (in mg kg^−1^, dry matter) of macro- and trace elements in the investigated mushrooms, including calculated LODs and LOQs (in mg kg^−1^), minimum (min), maximum (max), and average (avg) values.

	**Al**	**As**	**Ba**	**Be**	**Bi**	**Ca**	**Cd**	**Co**	**Cr**	**Cs**	**Cu**
LOD	2	0.003	0.05	0.001	0.001	15	0.002	0.002	0.03	0.002	0.03
LOQ	6	0.01	0.15	0.003	0.003	45	0.006	0.006	0.1	0.006	0.1
1/7256	7.83	0.019	0.096	<DL	<DL	26	0.681	0.006	0.313	0.016	2.19
1/7258	30.9	0.318	1.599	<DL	0.001	171	0.379	0.026	0.265	0.124	46.6
1/7259	19.4	0.394	0.518	<DL	0.004	187	0.356	0.125	0.419	0.002	38.6
1/7260	17.0	0.542	0.484	<DL	0.003	164	0.313	0.171	0.378	0.003	84.3
1/7262	33.2	0.482	1.367	<DL	0.001	273	0.144	0.031	0.246	0.015	28.8
1/7263	23.5	3.684	1.126	0.001	0.003	374	0.365	0.100	0.221	0.124	78.0
1/7267	24.4	0.549	0.528	<DL	0.010	146	0.271	0.092	0.198	0.017	67.2
1/7268	99.3	0.087	1.725	0.001	0.002	216	0.396	0.084	0.305	0.333	11.3
1/7269	12.6	0.219	0.307	<DL	0.002	188	0.384	0.033	0.279	0.008	56.6
1/7272	27.6	0.661	0.979	0.002	0.001	262	0.216	0.018	0.306	0.037	52.4
1/7273	43.9	0.151	0.591	0.001	0.001	231	0.490	0.139	0.343	0.604	20.0
1/7274	22.9	0.489	0.580	<DL	0.001	545	0.486	0.058	0.608	0.006	42.3
1/7275	40.7	0.008	0.810	<DL	<DL	223	0.116	0.021	0.159	0.035	9.44
1/7284	91.0	0.639	1.453	<DL	<DL	276	0.593	0.069	0.268	0.167	16.5
1/7288	34.4	0.260	0.554	<DL	0.002	257	0.550	0.048	0.337	0.581	8.85
1/7291	26.1	0.593	0.512	<DL	0.001	255	0.177	0.125	0.223	0.043	41.0
1/7292	77.2	0.012	0.793	0.002	0.001	256	0.549	0.701	0.761	1.956	32.9
1/7293	13.0	0.310	0.294	<DL	<DL	144	4.547	0.043	0.278	0.482	26.2
1/7294	16.0	<DL	0.227	<DL	<DL	128	3.956	0.011	0.230	0.419	44.8
avg	34.8	0.52	0.77	0.001	0.002	227	0.79	0.10	0.32	0.26	37.3
min	7.83	0.01	0.10	0.001	0.001	25.6	0.12	0.01	0.16	0.002	2.19
max	99.3	3.68	1.73	0.002	0.010	545	4.55	0.70	0.76	1.96	84.3
	**Fe**	**K**	**Li**	**Mg**	**Mn**	**Mo**	**Na**	**Ni**	**Pb**	**Rb**	
LOD	3	15	0.002	6	0.25	0.005	3	0.03	0.03	0.03	
LOQ	10	45	0.006	18	0.75	0.015	10	0.1	0.1	0.1	
1/7256	11.0	42327	0.019	1706	5.58	0.018	77.2	0.325	0.072	14.7	
1/7258	95.7	25183	0.031	1086	21.5	0.557	60.4	0.559	0.135	10.3	
1/7259	52.5	17100	0.015	1194	13.6	0.332	70.3	0.611	1.11	5.48	
1/7260	103	22924	0.012	1597	27.7	0.355	36.1	0.742	0.509	2.59	
1/7262	49.4	41252	0.026	1395	31.4	0.812	53.5	0.363	0.228	2.69	
1/7263	49.0	65291	0.025	2014	11.6	0.488	829	0.547	0.352	24.6	
1/7267	79.7	17410	0.013	1520	38.2	0.375	41.6	0.472	0.868	26.5	
1/7268	87.9	66745	0.078	2067	22.9	0.015	932	0.327	0.261	289	
1/7269	60.6	33029	0.017	1300	11.5	0.409	49.5	0.358	0.437	10.1	
1/7272	76.1	27369	0.016	959	36.4	0.606	47.9	0.663	0.128	40.1	
1/7273	67.4	80836	0.037	2093	25.6	0.016	653	0.247	0.406	185	
1/7274	57.0	49527	0.013	1639	14.5	0.164	835	0.181	0.161	27.9	
1/7275	32.9	38559	0.027	1060	21.3	0.045	410	0.187	0.122	27.3	
1/7284	62.7	34991	0.079	1248	8.13	0.007	60.6	0.508	0.180	144	
1/7288	29.7	25886	0.021	1153	4.93	0.010	35.9	0.505	0.187	191	
1/7291	48.0	31622	0.010	1031	37.6	1.145	72.5	0.432	0.535	37.6	
1/7292	77.3	46135	0.074	939	25.1	0.103	74.1	1.595	4.019	299	
1/7293	23.9	60912	0.025	1876	7.26	0.235	1011	0.162	0.299	46.8	
1/7294	22.1	43277	0.012	1694	5.87	0.050	25.2	0.259	0.234	133	
avg	57.1	40546	0.029	1451	19.5	0.302	283	0.476	0.539	79.8	
min	11.0	17100	0.010	939	4.93	0.007	25.2	0.162	0.072	2.59	
max	103	80836	0.079	2093	38.2	1.145	1011	1.595	4.019	299	
	**Sb**	**Sc**	**Se**	**Sn**	**Sr**	**Ti**	**Tl**	**U**	**V**	**Zn**	
LOD	0.002	0.002	0.02	0.003	0.5	0.5	0.001	0.001	0.002	1.5	
LOQ	0.006	0.006	0.06	0.01	1.5	1.5	0.003	0.003	0.006	4.5	
1/7256	0.002	<DL	<DL	0.095	0.052	0.52	<DL	<DL	0.011	17.3	
1/7258	0.015	<DL	0.144	0.094	0.386	2.89	0.001	<DL	0.153	74.2	
1/7259	0.006	0.099	0.725	0.195	0.394	1.15	<DL	0.010	0.094	143	
1/7260	0.006	0.047	0.788	0.140	0.391	0.65	0.001	0.005	0.081	109	
1/7262	0.013	0.031	0.842	0.156	0.734	2.16	0.005	0.004	0.131	100	
1/7263	0.068	<DL	1.593	0.118	1.198	2.29	0.003	0.004	0.083	97.6	
1/7267	0.004	<DL	0.679	0.086	0.282	1.20	<DL	<DL	0.068	127	
1/7268	0.012	0.012	<DL	0.085	0.966	9.70	0.006	0.002	0.174	89.4	
1/7269	0.005	0.026	0.387	0.107	0.309	1.91	0.003	<DL	0.052	72.8	
1/7272	0.007	0.011	0.254	0.116	0.609	1.69	0.001	0.001	0.111	66.9	
1/7273	0.003	0.012	<DL	0.117	0.497	3.51	0.011	0.001	0.083	69.8	
1/7274	0.011	0.004	0.386	0.083	0.756	1.57	0.003	0.003	0.054	58.3	
1/7275	0.004	<DL	<DL	0.102	0.428	3.58	0.001	<DL	0.067	45.8	
1/7284	0.002	0.024	0.414	0.068	0.734	8.65	0.052	0.008	0.138	133	
1/7288	0.003	0.013	0.318	0.122	0.479	2.73	0.129	0.003	0.038	103	
1/7291	0.004	<DL	0.205	0.057	0.318	1.18	0.004	<DL	0.136	58.3	
1/7292	0.004	0.094	<DL	0.114	0.543	7.76	0.002	0.005	0.150	64.2	
1/7293	0.035	<DL	1.49	0.066	0.277	0.93	0.007	0.001	0.018	75.3	
1/7294	0.003	<DL	<DL	0.069	0.488	1.51	0.007	0.001	0.025	91.5	
avg	0.011	0.034	0.633	0.105	0.518	2.93	0.012	0.003	0.088	84.1	
min	0.002	0.004	0.144	0.057	0.052	0.52	0.001	0.001	0.011	17.3	
max	0.068	0.099	1.593	0.195	1.198	9.70	0.129	0.010	0.174	143	

DL—detection limits.

**Table 3:** Concentration of REY (in mg kg^−1^, dry matter) in the investigated mushrooms, including calculated LODs and LOQs (in mg kg^−1^), minimum (min), maximum (max), and average (avg) values.

	La	Ce	Pr	Nd	Sm	Eu	Gd	Tb	Dy	Ho	Er	Tm	Yb	Lu	Y	∑REY
LOD	0.001	0.001	0.001	0.001	0.001	0.001	0.001	0.001	0.001	0.001	0.001	0.001	0.001	0.001	0.001	
LOQ	0.003	0.003	0.003	0.003	0.003	0.003	0.003	0.003	0.003	0.003	0.003	0.003	0.003	0.003	0.003	
1/7256	0.003	0.006	0.001	0.002	0.001	<DL	<DL	<DL	0.009	<DL	<DL	<DL	0.007	<DL	0.002	0.031
1/7258	0.012	0.028	0.003	0.012	0.003	0.001	<DL	<DL	0.011	0.001	0.001	<DL	0.008	<DL	0.012	0.092
1/7259	0.005	0.009	0.001	0.004	0.001	0.000	<DL	<DL	0.010	<DL	0.001	<DL	0.008	<DL	0.004	0.043
1/7260	0.006	0.010	0.001	0.005	0.001	0.001	<DL	<DL	0.011	<DL	0.001	<DL	0.008	<DL	0.007	0.051
1/7262	0.016	0.031	0.004	0.016	0.003	0.001	<DL	<DL	0.011	0.001	0.001	<DL	0.009	<DL	0.013	0.094
1/7263	0.021	0.039	0.006	0.022	0.006	0.001	0.002	0.001	0.013	0.001	0.002	<DL	0.008	<DL	0.024	0.146
1/7267	0.009	0.017	0.002	0.007	0.001	0.001	<DL	<DL	0.011	<DL	<DL	<DL	0.008	<DL	0.004	0.060
1/7268	0.053	0.115	0.013	0.057	0.010	0.002	<DL	0.001	0.016	0.002	0.003	0.001	0.010	0.001	0.032	0.316
1/7269	0.005	0.012	0.001	0.003	0.001	<DL	<DL	<DL	0.010	<DL	<DL	<DL	0.007	<DL	0.006	0.045
1/7272	0.015	0.026	0.003	0.012	0.002	0.001	<DL	<DL	0.011	<DL	0.001	<DL	0.008	<DL	0.009	0.088
1/7273	0.019	0.035	0.004	0.014	0.003	0.001	<DL	<DL	0.011	0.001	0.001	<DL	0.008	<DL	0.012	0.109
1/7274	0.010	0.019	0.003	0.010	0.003	0.001	<DL	<DL	0.010	<DL	0.001	<DL	0.007	<DL	0.006	0.070
1/7275	0.024	0.051	0.006	0.026	0.005	0.001	<DL	<DL	0.011	0.000	0.001	<DL	0.008	<DL	0.011	0.144
1/7284	0.093	0.137	0.017	0.063	0.011	0.002	<DL	0.002	0.020	0.003	0.006	0.001	0.014	0.001	0.082	0.452
1/7288	0.028	0.031	0.004	0.014	0.003	0.001	<DL	0.001	0.013	0.001	0.003	<DL	0.007	<DL	0.040	0.146
1/7291	0.016	0.024	0.003	0.014	0.003	0.001	<DL	<DL	0.008	<DL	0.001	<DL	0.006	<DL	0.009	0.085
1/7292	0.050	0.080	0.008	0.034	0.007	0.001	<DL	0.001	0.013	0.001	0.004	0.001	0.009	0.001	0.029	0.239
1/7293	0.008	0.013	0.001	0.006	0.002	<DL	<DL	<DL	0.010	<DL	<DL	<DL	0.007	<DL	0.004	0.051
1/7294	0.010	0.019	0.002	0.008	0.002	<DL	<DL	<DL	0.010	<DL	0.001	<DL	0.007	<DL	0.005	0.064
avg	0.021	0.037	0.004	0.017	0.004	0.001	0.002	0.001	0.012	0.001	0.002	0.001	0.008	0.001	0.016	0.122
min	0.003	0.006	0.001	0.002	0.001	<DL	<DL	<DL	0.008	<DL	<DL	<DL	0.006	<DL	0.002	0.031
max	0.093	0.137	0.017	0.063	0.011	0.002	0.002	0.002	0.02	0.003	0.006	0.001	0.014	0.001	0.082	0.452

DL—detection limits.

**Table 4:** Concentration of macro- and trace elements in different mushrooms from the region (expressed in mg/kg or * g/kg). If not otherwise specified, the results refer to the fruiting body.

	Croatia		Serbia			Italy	
Element	Edible (Caps and Stipes) [40,41,42]	*Agaricus* sp., *Trichaptum biforme* [12]	*M. procera* (Caps and Stipes) [34,35]	Edible [50]	Boletaceae (Edible) [51]	Different Fungal Species [38]	*Morchella* Group [37]
Al		623–925	29–5664	54.2–396	27.7–608		0.05–0.33
As		0.19–3.64	0.01–3.40		0.03–1.66	0.10–11.6	<DL–0.23
Ba		19.2–72.4	0.20–46	1.20–8.7			0.33–4.06
Be		0.02–0.41					<DL
Bi		0.04–0.08			0.02–1.40		<DL
Cd	0.60–3.23	0.02–0.18	0.04–43.5	0.27–2.93	0.68–2.94	0.16–101	0.18–21.5
Co		0.17–2.92	<DL–12.0		0.07–0.72		0.06–0.28
Cr	0.77–3.85	1.32–13.19	0.20–13.8	3.25–10.8	0.16–1.34		0.26–2.18
Cs		0.15–1.18					0.05–0.48
Cu	7.41–78.2	6.67–32.4	29–304	12.2–73.6	4.66–34.3		10.1–63
Fe	49.3–154	365–4905	30–4018	45.9–319	24.4–515		35–517
Li		0.49–7.57		0.21–1.27			0.03–0.67
Mn		18.9–248	7.6–367	9.0–35.5	2.91–23.8		7.5–46.3
Mo		0.16–0.43		1.14–2.3			0.2–0.45
Ni	2.02–4.10	1.02–7.16	0.09–11.7	2.24–5.04	0.40–1.69		0.6–2.33
Pb	0.48–1.91	1.29–5.73	<DL–14.3	6.32–9.8	0.29–10.6	0.58–10.6	<DL–1.09
Rb		2.28–12.6			23.74–500		2.55–36.6
Sb		0.05–0.28		4.9–26.1			0.01–0.05
Sc							
Se			0.17–3.3		0.04–2.32	0.2–94.4	<DL–0.49
Sn		0.14–0.61					<DL
Sr		7.98–21.7	0.08–29.9	1.15–4.34	0.50–4.71		0.8–5.8
Ti		59.02–647	1.2–156				6.31–18.8
Tl		0.01–0.17					0.002–0.13
You		0.04–0.46					0.001–0.02
V		1.34–15.7					0.06–1.26
Zn	41.99–90.56	21.3–59.1	27.3–535	38.7–117	17.6–301.6		99.7–259
Ca *			0.02–4.43	0.55–2.12	0.11–2.33		0.28–3.51
K *			11–120	11.3–41.8	10.1–21.2		25.6–> 43
Mg *			0.69–3.4	0.29–1.12			0.79–2.64
Na *			11–1900	0.41–0.93	39.8–916		0.1–0.83

**Table 5:** The comparison of the determined REY concentration with the literature values for mushrooms from different regions (all expressed in mg/kg). If not otherwise specified, the results refer to the fruiting body.

	Croatia	Poland			Czech Republic	Italy
Element	*Agaricus* sp. (Caps and Stipes) [44]	*Agaricus* sp., *Trichaptum biforme* [12]	*M. procera* (Caps and Stipes) [34,35]	Edible [48]	*Boletaceae* (Edible) [49]	Different Fungal Species [38]
La	1.373	0.083 ± 0.049	0.06	<0.01–0.08	0.023	0.015–0.488
Ce	4.137	0.18 ± 0.091	0.12		0.042	0.025–0.843
Pr	0.526	0.017 ± 0.009	0.04	0.02–1.8	0.0056	<DL–0.109
Nd	2.137	0.058 ± 0.003	0.17	0.11–0.45	0.020	0.011–0.446
Sm	0.467	0.012 ± 0.006	0.03		0.0041	0.0021–0.0822
Eu	0.099	0.0027 ± 0.018	<0.01		0.00068	<DL–0.019
Gd	0.305	0.011 ± 0.006	0.05	<0.01–0.05	0.0023	0.002–0.081
Tb	0.057	0.0018 ± 0.0009	0.03		0.00059	<DL–0.012
Dy	0.285	0.010 ± 0.005	<0.01		0.0022	0.001–0.0724
Ho	0.062	0.0023 ± 0.0012	0.04	<0.01–0.30	0.00042	0.0003–0.015
Er	0.163	0.0070 ± 0.0035	0.72		0.0013	0.0005–0.0429
Tm	0.027	0.0011 ± 0.0006	0.03		0.00017	0.0001–0.006
Yb	0.150	0.0073 ± 0.0037	0.02		0.0013	<DL–0.041
Lu	0.027	0.0011 ± 0.0005	0.02		0.00013	<DL–0.0058
Y	0.693	0.074 ± 0.039	0.04			0.009–0.549
∑REY	9.797	0.481	0.23		0.104	

**Table 6:** Non-carcinogenic risk data (expressed by health risk indexes THQ and HI) obtained in the health risk assessment of metals through the consumption of the edible wild mushrooms from Croatia. The RfD data are expressed as µg kg^−1^ day^−1^.

	Target Hazard Quotient (THQ)													Hazard Index (HI)
	As	Ba	Be	Cd	Cu	Fe	Mn	Ni	Pb	Sb	Sr	U	Zn	
1/7256	0.026	0.000	0.000	0.280	0.023	0.006	0.016	0.007	0.008	0.002	0.000	0.000	0.024	0.392
1/7258	0.436	0.003	0.000	0.156	0.479	0.056	0.063	0.011	0.016	0.015	0.000	0.000	0.102	1.337
1/7259	0.540	0.001	0.000	0.146	0.397	0.031	0.040	0.013	0.130	0.006	0.000	0.001	0.196	1.501
1/7260	0.742	0.001	0.000	0.129	0.866	0.060	0.081	0.015	0.060	0.006	0.000	0.001	0.149	2.111
1/7262	0.660	0.003	0.000	0.059	0.296	0.029	0.092	0.007	0.027	0.013	0.001	0.001	0.137	1.325
1/7263	5.047	0.002	0.000	0.150	0.801	0.029	0.034	0.011	0.041	0.070	0.001	0.001	0.134	6.321
1/7267	0.752	0.001	0.000	0.111	0.690	0.047	0.112	0.010	0.102	0.004	0.000	0.000	0.174	2.004
1/7268	0.119	0.004	0.000	0.163	0.116	0.052	0.067	0.007	0.031	0.012	0.001	0.000	0.122	0.694
1/7269	0.300	0.001	0.000	0.158	0.582	0.036	0.034	0.007	0.051	0.005	0.000	0.000	0.100	1.273
1/7272	0.905	0.002	0.000	0.089	0.538	0.045	0.107	0.014	0.015	0.007	0.000	0.000	0.092	1.815
1/7273	0.207	0.001	0.000	0.201	0.205	0.040	0.075	0.005	0.048	0.003	0.000	0.000	0.096	0.882
1/7274	0.670	0.001	0.000	0.200	0.435	0.033	0.043	0.004	0.019	0.011	0.001	0.000	0.080	1.496
1/7275	0.011	0.002	0.000	0.048	0.097	0.019	0.063	0.004	0.014	0.004	0.000	0.000	0.063	0.324
1/7284	0.875	0.003	0.000	0.244	0.170	0.037	0.024	0.010	0.021	0.002	0.001	0.001	0.182	1.570
1/7288	0.356	0.001	0.000	0.226	0.091	0.017	0.014	0.010	0.022	0.003	0.000	0.000	0.141	0.883
1/7291	0.812	0.001	0.000	0.073	0.421	0.028	0.110	0.009	0.063	0.004	0.000	0.000	0.080	1.602
1/7292	0.016	0.002	0.000	0.226	0.338	0.045	0.074	0.033	0.472	0.004	0.000	0.001	0.088	1.299
1/7293	0.425	0.001	0.000	1.869	0.269	0.014	0.021	0.003	0.035	0.036	0.000	0.000	0.103	2.776
1/7294	0.000	0.000	0.000	1.626	0.460	0.013	0.017	0.005	0.027	0.003	0.000	0.000	0.125	2.278
RfD	0.3 ^a^	200 ^a^	2 ^a^	1 ^a^	40 ^b^	700 ^b^	140 ^a^	20 ^a^	3.5 ^b^	0.4 ^a^	600 ^a^	3 ^a^	300 ^a^	

^a^—IRIS assessments (USEPA) [49]. ^b^—from Dowlati et al. [48].

## Data Availability

Not applicable.

## Supplementary Materials

The following are available online at https://www.mdpi.com/article/10.3390/jof7121068/s1, Table S1: title Spearman correlation coefficients of the investigated elements in mushrooms. Correlation coefficients marked in red are significant at *p* < 0.05.
