# Supplementary material for: Distribution and Origin of Major, Trace and Rare Earth Elements in Wild Edible Mushrooms: Urban vs. Forest Areas
Source: J Fungi (Basel). 2021 Dec 12;7(12):1068. doi: 10.3390/jof7121068 (PMC8706631; doi:10.3390/jof7121068)
Supplement: Supplementary file 1 [file jof-07-01068-s001.zip › jof-1433981-supplementary.pdf]

Table S1. Spearman correlation coefficients of the investigated elements in mushrooms. Correlation coefficients marked in red are significant at $p<0.05$.

	As	Ba	Be	Bi	Ca	Cd	Co	Cr	Cs	Cu	Fe	K	Li	Mg	Mn
**Al**	-0.22	**0.81**	**-0.48**	-0.06	**0.52**	-0.16	0.28	-0.02	**0.50**	-0.39	0.40	0.18	**0.69**	-0.25	0.32
**As**		-0.01	0.14	0.23	0.21	-0.13	-0.01	-0.32	-0.15	**0.61**	0.01	-0.23	**-0.50**	0.02	0.14
**Ba**			-0.45	-0.02	**0.62**	-0.32	0.09	-0.14	0.21	-0.09	**0.50**	0.14	**0.67**	-0.16	0.32
**Be**				0.25	-0.32	-0.03	-0.37	-0.21	**-0.50**	0.01	-0.34	**-0.58**	-0.45	-0.27	-0.26
**Bi**					-0.26	0.07	-0.08	-0.45	0.04	0.11	-0.09	-0.14	0.06	0.14	-0.29
**Ca**						-0.25	0.22	0.09	0.08	-0.01	0.10	0.21	0.27	-0.23	0.17
**Cd**							-0.11	0.36	**0.53**	-0.36	-0.30	0.43	0.25	0.41	**-0.73**
**Co**								0.36	0.10	0.22	**0.47**	0.01	0.02	0.04	0.39
**Cr**									-0.04	-0.13	0.25	0.05	0.04	0.00	-0.11
**Cs**										-0.36	-0.09	**0.51**	**0.53**	0.08	-0.19
**Cu**											0.44	-0.32	**-0.54**	-0.05	0.40
**Fe**												-0.23	0.19	-0.12	**0.62**
**K**													**0.46**	**0.60**	-0.21
**Li**														0.07	-0.15
**Mg**															-0.25

	Mo	Na	Ni	Pb	Rb	Sb	Se	Sn	Sr	Ti	Tl	U	V	Zn	∑REE
**Al**	-0.33	0.14	0.19	-0.06	**0.56**	-0.15	0.07	0.04	**0.58**	**0.87**	0.35	-0.06	**0.71**	0.06	**0.82**
**As**	0.42	-0.39	0.26	0.07	-0.16	0.15	-0.41	-0.14	0.24	-0.29	0.08	0.07	0.04	0.43	-0.15
**Ba**	0.00	0.21	0.27	-0.24	0.19	0.32	-0.10	0.13	**0.70**	**0.80**	0.15	0.11	**0.77**	0.12	**0.81**
**Be**	0.13	-0.40	-0.15	-0.18	-0.46	-0.12	-0.40	-0.21	**-0.56**	**-0.50**	-0.16	0.19	-0.43	0.15	-0.39
**Bi**	-0.20	-0.15	-0.09	0.08	-0.04	0.11	0.18	-0.11	-0.04	0.06	0.14	0.01	-0.20	**0.58**	0.05
**Ca**	0.07	0.18	0.18	-0.12	0.21	0.12	-0.28	0.18	**0.77**	**0.52**	0.31	-0.19	0.04	-0.03	**0.54**
**Cd**	**-0.64**	0.15	-0.28	-0.10	**0.51**	-0.31	0.28	-0.35	-0.06	0.00	**0.47**	0.14	-0.37	0.00	-0.1
**Co**	-0.02	0.18	0.39	**0.80**	0.13	-0.01	0.05	0.22	0.18	0.10	0.08	0.13	0.41	0.32	0.32
**Cr**	-0.30	0.09	0.22	0.16	0.16	-0.12	0.02	0.34	0.11	-0.07	-0.05	0.17	0.02	-0.07	0.10
**Cs**	-0.43	0.17	-0.08	0.03	**0.85**	-0.23	0.29	-0.27	0.22	**0.52**	**0.65**	-0.20	0.14	-0.08	0.35
**Cu**	**0.69**	-0.36	0.42	0.40	-0.46	0.44	-0.36	0.12	-0.02	-0.31	-0.33	0.08	0.07	0.24	-0.22
**Fe**	0.16	-0.12	**0.53**	0.29	-0.05	0.23	-0.22	0.17	0.21	0.32	-0.22	0.27	**0.66**	0.23	0.39
**K**	-0.34	**0.70**	**-0.56**	-0.12	**0.49**	0.09	**0.62**	-0.25	**0.45**	0.34	**0.48**	-0.35	-0.05	-0.40	0.03
**Li**	-0.46	0.44	-0.04	-0.23	0.43	0.01	0.34	0.02	0.39	**0.80**	0.37	0.08	0.44	-0.04	**0.68**
**Mg**	-0.30	0.39	**-0.53**	0.01	0.01	0.15	**0.50**	-0.09	0.12	-0.14	0.3	0.04	-0.33	0.13	-0.33

	Mo	Na	Ni	Pb	Rb	Sb	Se	Sn	Sr	Ti	Tl	U	V	Zn	∑REE
**Mn**	**0.55**	-0.07	0.31	0.34	-0.14	0.20	-0.18	0.11	0.08	0.04	-0.33	-0.07	**0.57**	-0.04	0.05
**Na**		-0.19	0.31	0.22	**-0.60**	**0.56**	**-0.48**	0.14	-0.17	-0.38	-0.38	-0.05	0.20	-0.04	-0.25
**Ni**			-0.45	-0.06	0.23	0.30	0.37	-0.27	0.21	0.19	0.09	-0.15	0.10	-0.43	0.09
**Pb**				0.34	-0.16	0.06	-0.33	**0.48**	0.12	0.07	-0.31	**0.47**	**0.53**	0.40	**0.48**
**Rb**					-0.01	0.04	0.07	0.16	-0.13	-0.15	-0.02	0.04	0.20	0.38	-0.01
**Sb**						-0.36	0.24	-0.39	0.35	**0.53**	**0.56**	-0.29	0.15	-0.17	0.33
**Se**							-0.16	0.14	0.19	-0.06	-0.15	0.05	0.19	0.11	0.03
**Sn**								0.08	0.14	0.15	0.05	-0.05	-0.17	-0.14	-0.02
**Sr**									0.17	0.01	-0.24	0.19	0.05	0.30	0.25
**Ti**										**0.63**	0.35	-0.11	**0.46**	0.12	**0.62**
**Tl**											0.41	-0.16	**0.57**	-0.02	**0.82**
**U**												-0.37	0.03	0.19	0.26
**V**													0.14	0.44	0.15
**Zn**														0.14	**0.73**
**∑REE**															0.24
